# Experimental validation of immunogenic SARS-CoV-2 T cell epitopes identified by artificial intelligence

**DOI:** 10.3389/fimmu.2023.1265044

**Published:** 2023-11-17

**Authors:** Lorenzo Federico, Brandon Malone, Simen Tennøe, Viktoriia Chaban, Julie Røkke Osen, Murat Gainullin, Eva Smorodina, Hassen Kared, Rahmad Akbar, Victor Greiff, Richard Stratford, Trevor Clancy, Ludvig Andre Munthe

**Affiliations:** ^1^ Department of Immunology, Oslo University Hospital, Oslo, Norway; ^2^ KG Jebsen Centre for B cell Malignancies, Institute of Clinical Medicine, University of Oslo, Oslo, Norway; ^3^ NEC OncoImmunity AS, Oslo, Norway; ^4^ Institute of Clinical Medicine, Oslo University Hospital, Oslo, Norway

**Keywords:** T cell, COVID-19, CD8+ lymphocytes, CD4+ lymphocytes, vaccine, SARS-CoV-2, artificial intelligence, antigen (Ag)

## Abstract

During the COVID-19 pandemic we utilized an AI-driven T cell epitope prediction tool, the NEC Immune Profiler (NIP) to scrutinize and predict regions of T cell immunogenicity (hotspots) from the entire SARS-CoV-2 viral proteome. These immunogenic regions offer potential for the development of universally protective T cell vaccine candidates. Here, we validated and characterized T cell responses to a set of minimal epitopes from these AI-identified universal hotspots. Utilizing a flow cytometry-based T cell activation-induced marker (AIM) assay, we identified 59 validated screening hits, of which 56% (33 peptides) have not been previously reported. Notably, we found that most of these novel epitopes were derived from the non-spike regions of SARS-CoV-2 (Orf1ab, Orf3a, and E). In addition, *ex vivo* stimulation with NIP-predicted peptides from the spike protein elicited CD8^+^ T cell response in PBMC isolated from most vaccinated donors. Our data confirm the predictive accuracy of AI platforms modelling *bona fide* immunogenicity and provide a novel framework for the evaluation of vaccine-induced T cell responses.

## Introduction

The rapid deployment of SARS-CoV-2 vaccines mitigated global hospitalization rates and is estimated to have saved between 14 and 20 million lives during the first year of vaccination (12). However, although over 13 billion doses have been administered to date and ~70% of the world’s population has received at least one dose (13), the limited capacity of current vaccines to fully suppress infectivity raises concerns about emerging variants (14).

Because CD8^+^ T cells are vital in forging protective immunity against viruses and can control COVID-19 infection in the early stages, even in the absence of a serological response (7, 15–17), we sought to develop a refined approach for the characterization of epitopes capable of eliciting cell-mediated immune response to aid in the development of T cell vaccines.

The NEC Oncoimmunity Immune Profiler (NIP) is an example of an AI algorithm that employs a unique machine learning approach to predict T cell immunogenicity for any Human Leukocyte Antigen (HLA) allele. The NIP algorithm has previously been used to analyze the SARS−CoV−2 proteome to delineate universal blueprints for potential COVID-19 T cell-based vaccines (1). This involved scanning all possible minimal epitopes of length 9 and 10 amino acids in the SARS−CoV−2 proteome and in predicted regions of T cell immunogenicity (hotspots) that could potentially be used as universally protective T cell vaccine candidates, or as biomarkers to monitor T cell responses induced during infection or vaccination. Compared to other algorithms, the NIP platform is expected to identify *bona fide* T cell antigen hotspots because it predicts the immunogenicity of antigens not only based on HLA-peptide binding affinity, but also based on features that determine the propensity of an antigen to undergo intracellular processing and be presented on the cell surface.

From all the SARS−CoV−2 hotspots identified by the NIP algorithm (1), we selected a group of 101 peptides with lengths of 9 to 10 amino acids predicted to preferentially stimulate response in CD8^+^ T cells (18). Although the relevance of non-spike regions in SARS-CoV-2 evolution has not been as extensively investigated as done for the S protein (19, 20), the peptide selection used in this study included a significant number of epitopes from those regions because their inclusion in vaccines is expected to broaden and boost protective T cell memory response (21). Using flow cytometry and PBMC isolated from previously infected and/or vaccinated patients, we tested the antigenicity of these peptides by quantifying the expression of activation-induced markers (AIMs) CD137, CD40L, IFNγ, and TNF as previously shown (2–4). Moreover, to overcome the limitations of single-population analysis, we adopted a multimarker-based data analysis strategy which allowed us to capture the full spectrum of the T cell response. This approach not only confirmed preferential activation of CD8^+^ T cells by NIP peptides but also helped identify novel SARS-CoV-2 immunogenic epitopes, the majority of which belonged to non-spike regions. Subsequent testing of healthy individuals who received 2 or 3 doses of COVID-19 vaccine showed that NIP peptides elicited CD8^+^ T cell activation *ex vivo*, indicating that these epitopes can be used as a complementary screening tool for the assessment of cell-mediated immune response.

Altogether, our data suggest that these antigens not only have the potential to control viral infection and spreading if used in a vaccine but may also provide the groundwork for the formulation of diagnostic tools specifically designed to monitor CD8^+^ T cell response and infection during the post-pandemic period, when new variants are expected to emerge.

## Materials and methods

### Patients

T cell reactivity to NIP-predicted peptides was validated *ex vivo* using PBMC isolated from blood collected between July 2^nd^, 2022, and November 3^rd^, 2022, from 12 healthy donors who received ≥ 2 doses of (mRNA) vaccines (Moderna/mRNA-1273 or Pfizer/BioNTech BNT162b2) and have contracted COVID-19 infection. Blood was collected one more time from a subgroup of subjects for peptide deconvolution experiments. Informed consent was obtained from all donors and approved by the Health Region South-East Regional Ethics committee.

### Reagents

Spike-C (PepTivator® SARS-CoV-2 Prot_S Complete; Miltenyi, # 130-127-953), and Spike-I (PepTivator® SARS-CoV-2 Prot_S; Militenyi, # 130-126-700) pools are collections of lyophilized 15-mers peptides with 11 aa overlaps spanning either the immunodominant regions (Spike-I pool sequence domains: aa 304-338, 421-475, 492-519, 683-707, 741-770, 785-802, and 885-1273), or the entire length (Spike-C pool sequence: aa 5-1273) of the SARS-CoV-2 spike glycoprotein (Protein QHD43416.1, GenBank MN908947.3). The full list of NIP peptides, including their length and corresponding protein, is reported in **Supplementary Table S1** (1). Peptides were synthesized by GenScript (Piscataway, NJ, USA) at a purity ≥85% and stored at a final concentration of 1.5mg/mL. Peptide solubilities are reported in **Supplementary Table S1**.

### PBMC isolation and biobanking

PBMC were isolated from whole blood using CPT tubes (BD vacutainer, # 362782) according to manufacturer instructions. Briefly, blood was spun at room temperature (1600g x 25 minutes) to separate PBMC, which were then transferred into a 50mL tube, washed in cold PBS (Gibco, # 10010-015), counted, and resuspended in FBS (Gibco, # 10270-106) complemented with 10% DMSO. Cells (≥ 5 million per cryogenic tube) were transferred in liquid nitrogen for long-term storage after overnight pre-chilling at -80°C in Mr. Frosty freezing containers (Nalgene™, # 5100-0001).

### T cell activation assay

Quantification of T cell activation was performed by flow cytometry as previously described (2–4). Briefly, PBMC were thawed, washed twice, and resuspended in RPMI 1640 medium with GlutaMAX™ supplement (Thermo Fisher Scientific, # 61870-010), 1mmol/L Sodium Pyruvate (Gibco # 11360-039), 1mmol/L MEM NEAA (Gibco # 11140-035), 50nmol/L 1-thioglycerol (Sigma-Aldrich, # M1753), 12μg/mL Gentamycin (VWR, # E737), and 10% heat-inactivated Foetal Bovine Serum (Gibco, # 10270-106). Cells were stimulated for 3 hours with Spike-I or Spike-C pools (8μl each), or NIP pools at a final concentration of 1.5μg/mL per peptide in a 96-well round bottom cell culture plate (1M cells in 200μL/well). After further 18h incubation with Brefeldin A/Monensin cocktail (GolgiStop 500X, Invitrogen # 00-4980-93), cell pellets were washed once in cold PBS and stained for flow cytometry. To test the performance of the pool, T cell activation was performed as described before. Briefly, PBMC were washed in cold RPMI 1640 medium with GlutaMAX™ supplement before undergoing live cell enrichment in magnetic columns according to manufacturer instructions (MACS MultiStand, # 130-042-303 with OctoMACS™ Separator). PBMC were distributed on a 96-well round bottom cell culture plate (200μL/well) at 10 million cells per mL in TexMACS medium (Miltenyi, # 130-096-197) supplemented with 1mmol/L Sodium Pyruvate (Gibco # 11360-039), 1mmol/L MEM NEAA (Gibco # 11140-035), 50nmol/L 1-thioglycerol (Sigma-Aldrich, # M1753), 12μg/mL Gentamycin (VWR, # E737), and 20U/mL IL-2 (R&D # AFL202). After 3h incubation, cells were washed, treated with appropriate stimuli in presence of anti-CD28/CD49d co-stimulatory antibodies (1:200 dilution; BD # 347690), and incubated for 1h. After an additional 18h incubation with Brefeldin A/Monensin cocktail, cells were harvested for flow cytometry processing.

### Flow cytometry

Pellets were washed in FACS Wash Buffer [PBS1X w/o Ca++ & Mg++ (Gibco # 10010023) supplemented with 1% bovine serum albumin (VWR, #K719)] and stained in 150μL of cold PBS containing Fixable Near IR Live/Dead viability stain (Molecular Probes, # L34976) for 10 minutes in the dark. Cells were then washed one time in cold PBS1x and then resuspended in 50µL of FACS Wash Buffer containing the following fluorochrome-conjugated antibodies: BV605 anti-human CD3 (Clone SK7; BD Biosciences, # 563219), PerCP-Cy5.5 anti-human CD4 (Clone OKT4; Biolegend, # 317428), and Alexa Fluor 488 anti-human CD8 (Clone OKT8; Invitrogen, # 53-0086-42). After 30 minutes incubation on ice, cells were washed once and permeabilized for 30 minutes at room temperature in 150µL BD Cytofix/Cytoperm solution and washed two times in 200µL of 1x BD perm/wash solution (BD Biosciences Fixation and permeabilization kit; # 554714). To increase detection sensitivity (5), we detected AIM markers by staining after permeabilization. Cells were resuspended in 50µL of 1x BD perm/wash solution containing the following fluorochrome-conjugated antibodies: APC anti-human CD137 (Clone 4B4-1; BD Biosciences, # 550890), BV711 anti-human CD40L (Clone 24-31; Biolegend, # 310837), PE anti-human IFNγ (Clone 4S.B3; Biolegend, # 502509), and BV421 anti-human TNFA (Clone MAb11; BD Biosciences, # 562783). Antibody concentration was adjusted according to manufacturer instructions. After 25-minute incubation in the dark, cell pellets were washed once in PBS, resuspended, and acquired on an Attune NxT Flow Cytometer (Thermo Fisher). For pool performance experiments, stimulated cells were washed once in 1xPBS containing 5% FBS and 0.1% sodium azide, and then stained in the dark for 10 minutes with 0.5μL of Fixable Near IR Live/Dead viability stain (Molecular Probes, # L34976) in a final volume of 10μL cold PBS containing 5% FBS. Cells were then permeabilized at 4°C for 20 minutes in 100μL BD Cytofix/Cytoperm solution (BD # 554714), washed twice in 200μL of 1x BD perm/wash solution, and stained in 20µL of 1x BD perm/wash solution containing the following fluorochrome-conjugated antibodies: AF488 anti-human IL-2 (Clone MQ1-17H12; BioLegend, # 500314), PerCP-Cy5.5 anti-human CD8 (Clone RPA-T8; BioLegend, # 301032), PE anti-human CD137 (Clone 4B4-1; BioLegend, # 309804), PE-CF594 anti-human Granzyme B (Clone GB11; BD # 562462), PE-Cy5 anti-human (CD4 Clone RPA-T4; BD # 566925), PE-Cy7 anti-human TNF (Clone MAb11; Invitrogen, # 25-7349-82), AF647 anti-human IFNγ (BioLegend, #502516), BV510 anti-human CD40L (Biolegend, #310830), BV605 anti-human CD3 (Clone SK7; BD # 563219), and BV421 anti-human Perforin 1 (Clone dG9; BioLegend # 308122). BV711 anti-human CD107a antibody (Clone H4A3; BioLegend, # 328640) was added at the time of cell stimulation. Antibody concentration was adjusted according to manufacturer instructions. After 30-minute incubation in the dark, the cell pellet was washed twice in BD Perm/Wash™ buffer, resuspended, and acquired.

### Evaluation of T cell phenotype

T cell reactivity was evaluated using the reactivity score (RS). RS values were generated as previously reported (4) from the analysis of cell populations defined by the combined expression of 4 AIMs normally used to quantify T cell activation (3, 6–8). These markers and 3 gating areas of interest (Single +, Double +, and All) were used to define the 16 partially overlapping T cell populations listed in **Supplementary Table S3**. Out of the 16 T cell populations, we selected those whose response (frequency after stimulus minus background frequency) was informative in determining the degree of vaccine-induced response in healthy donor or immunocompromised patients, as previously described (4). The response frequency for each population was normalized by the average frequency of all measurements made for that population. We assigned a value of ‘zero’ to populations whose post-stimulation frequency was lower than their respective background frequency. The RS was then independently computed for each reactivity pattern shown in **Supplementary Figure S3** by averaging the normalized frequency of the specific populations that defined each pattern. An empirical threshold of 0.2 RS units was chosen as the level below which the response was considered low or absent. The compounded scores were calculated by adding the RS of each pattern.

### AI-driven antigen presentation and immunogenicity prediction

The immunogenicity of the peptides was predicted using the NEC Immune Profiler (NIP), an AI platform composed of several proprietary T cell epitope machine-learning prediction algorithms. The AI platform considers 1) the binding affinity of the peptide for the HLA alleles using 43 separate machine learning predictors that compute IC50 (nM) scores in an ensemble model, and 2) the antigen processing by the antigen processing machinery (APM) of the host infected cell. An ensemble of 12 Support Vector Machines and one neural network included in NIP and trained on validated mass spectrometry immunopeptidome datasets determined which peptides have the optimal features to be efficiently processed by the APM. The binding affinity scores, and the antigen processing scores are then used to predict the antigen presentation (AP) potential of each candidate peptide based on an ensemble machine learning model trained on a large proprietary database consisting of 10’s of millions of mass spec immunopeptidome datapoints. Finally, a bioinformatics assessment is made to assess the “difference from self” properties of the candidate peptides whereby each peptide is queried against the human self-proteome to capture the degree of “foreignness” of the peptide and increase the likelihood of identifying peptides that trigger a reactive T cell that is not tolerized in the T cell repertoire of the patient/donor. This score was termed here as the immune presentation potential (IP).

### Peptide selection

A previously-described peptide:HLA immunogenicity prediction tool (1) was used to identify candidate immunogenic peptides for validation. Briefly, predictions were made for each 9-mer and 10-mer candidate from the original Wuhan SARS-CoV-2 reference sequence (GenBank MN908947.3, downloaded April 15th, 2020). Both AP and IP predictions were made for each peptide and HLA from among the 100 most common HLAs. Based on these predictions, a set of “hotspots” enriched in predicted immunogenic peptides were identified based on either AP, IP, or both. Further, hotspots were designated as either “filtered” or “unfiltered” according to their conservation in the GISAID database. From among these hotspots, two sets of peptides were initially selected for validation. The selection was based specifically on 15 Class-I HLA alleles common in the Norwegian population. A first set of 66 peptides for validation was selected from any of the hotspots which passed the conservation filtering. These peptides could originate from any protein, but they all exceed stringent thresholds of (AP > 0.7) and (IP > 0.6) for at least one Norwegian allele. A second set of 35 peptides were selected specifically from hotspots in the spike protein. These hotspots were not required to pass the conservation filtering. The same AP and IP thresholds were used. Percentile rank scores (9), where lower values are better, were indicated for each peptide-HLA combination. We note that these percentile rank scores are only for presentation purpose; they were not used for epitope selection.

### Cross-reactivity to seasonal CoVs

We identified cross-reactive peptides to seasonal human CoV (sCoV) by using a two-step alignment approach. We first constructed a BLAST database of all complete human sCoV genome sequences for 229E (taxon ID # 11137), OC43 (taxon ID # 31631), NL63 (taxon ID # 277944), and HKU1 (taxon ID # 290028) downloaded from NCBI on July 5^th^, 2022. A loose BLASTP search (BLASTP search parameters: evalue = 100000, word_size = 2, qcov_hsp_perc = 75, gapopen = 6, gapextend = 2, comp_based_stats = F) was first used to align each peptide to all sequences in the database. As a second filtering step, a normalized BLOSUM-based similarity measure (10) was calculated between each peptide and the local alignment found with BLASTP. All alignments with a score less than 0.75 were removed. Peptides with less than 100 remaining alignments in the database were filtered. We consider any peptide with at least 20 alignments to the genomes of a given species to be cross-reactive with that species. We note that this step removes any peptides which align to regions that are not highly conserved across the different genomes.

### The immune epitope database

Literature-curated data were retrieved from IEDB (11) on May 5^th^, 2023. At this time the IEDB database reported a total of 3037 curated SARS-CoV-2 linear T cell epitopes experimentally shown to activate human T cells *ex vivo*.

### Statistical analysis

Statistical analyses were performed using GraphPad Prism V.8 (GraphPad software). Wilcoxon matched pairs signed rank test and Mann-Whitney U Two-tailed test were used where appropriate. For Principal Component Analysis (PCA) and plots loading, data were first standardized and the top two principal components by eigenvalues were selected for representation.

## Results

### Selection of NEC immune profiler SARS-CoV-2 immunoreactive epitopes

To identify CD8^+^ T cell epitopes relevant in vaccine-induced responses, we validated and characterized a group of 9 to 10 amino acid-long peptides identified by the NEC Immune Profiler (NIP) algorithm (**Supplementary Table S1**). In a previous study (1) the NIP AI platform was used to identify T cell immunogenic hotspots from all minimal epitopes screened in a moving window across the entire SARS-CoV-2 proteome. These hotspots were predicted by the AI to be universally protective against most Class I HLA alleles in the human population. Due to the source of the PBMC samples (Norway), we selected from these hotspots the top-ranked peptides for the 15 most frequent alleles in the Norwegian population. The list included the following alleles: HLA-A*01:01, HLA-A*02:01, HLA-A*03:01, HLA-A*23:0, HLA-A*29:02 HLA-B*07:02, HLA-B*08:01, HLA-B*15:01, HLA-B*15:02, HLA-B*40:01, HLA-B*44:02, HLA-C*03:03, HLA-C*04:01, HLA-C*07:01, and HLA-C*07:02 (**Supplementary Table S1).** The frequency information of the HLA alleles in the Norwegian population was derived from the Allele Frequency Net Database (AFND) (22). Of 101 peptides 43 belonged to the spike protein and 58 to non-spike regions, including the envelope protein (N = 2), the Orf1ab (N = 49), and the Orf3a (N = 7). It is important to note that the HLA genotype of the donors was not known at the time of the peptide selection process, and in some cases the donors were likely to be non-ethnic Norwegians. Therefore, the AI algorithm was blind to the HLA status of the patients to assess the universal nature of the candidate immunogenic epitopes. The rationale for peptide selection and the features of NIP-predicted peptides are described in Methods and in **Supplementary Table S1**, respectively.

### Peptide screening by single population analysis

**Figure 1:**
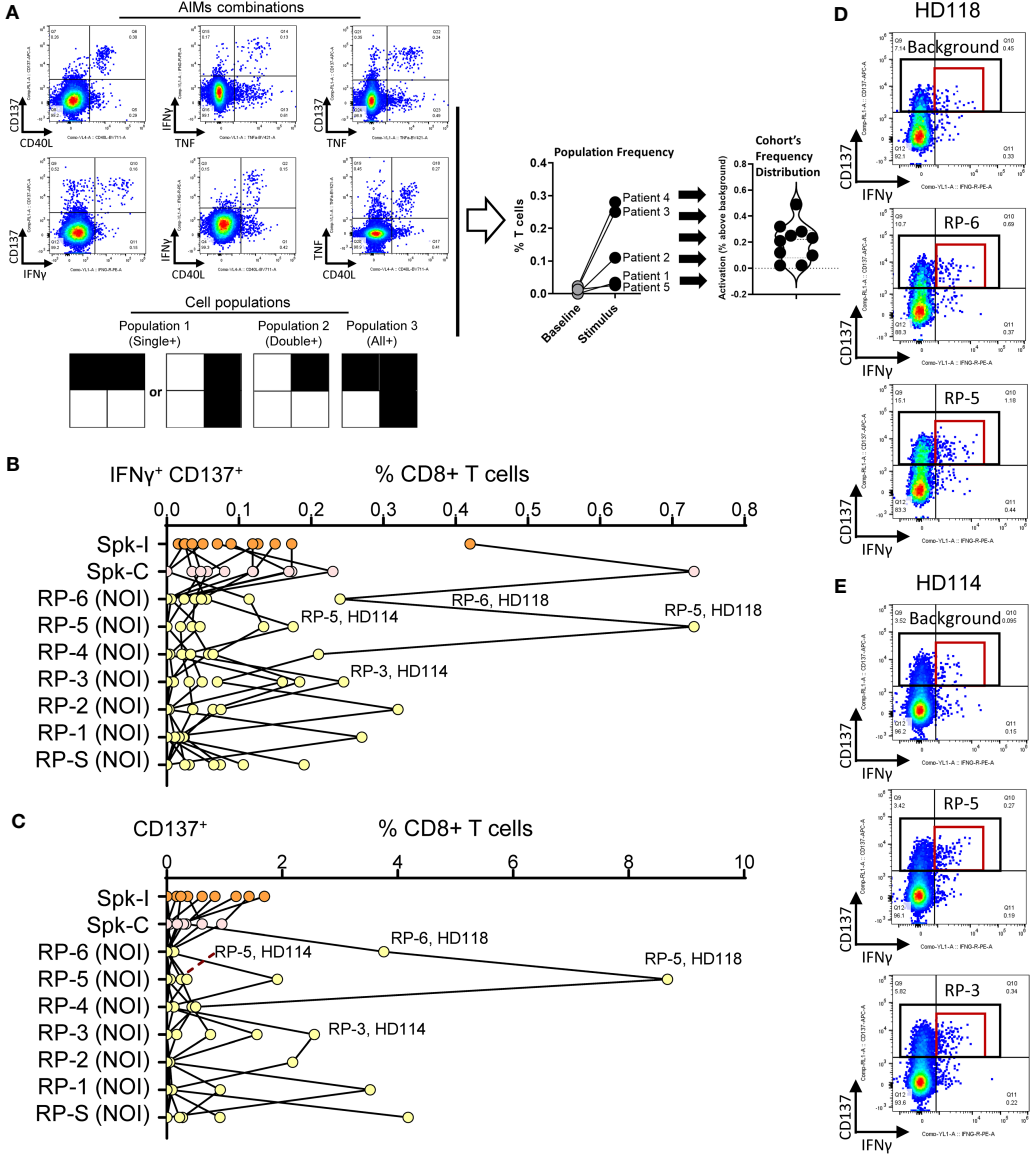
Screening hit identification by single population analysis. **(A)** AIM combinations and gated areas of interest (black squares) defining the 16 partially overlapping cell populations evaluated in the study. The magnitude of response is defined for each population as the frequency of the stimulation signal (stimulus) minus background frequency (baseline). **(B, C)** Degree of activation for **(B)** IFNγ^+^ CD137^+^ and **(C)** CD137^+^ CD8^+^ T cell populations in donors stimulated with Peptivator mixes (Spk-I and Spk-C) or random NOI’s protein pools (NOI RP). **(D)** Representative flow data for the activated T cell populations taken from panels B and C (red and black rectangles, respectively). The top responses for patients HD118 (RP-6, RP-5) and HD 114 (RP-5, RP-3) are shown.

T cells were stimulated with NIP 9-mer/10-mer peptides (random pools) or with overlapping 15-mer pools (Peptivator Spike-I or Spike-C, see Methods) and reactivity assessed using the flow cytometry-based AIM (Activation-Induced Marker) assay (see “Evaluation of T cell phenotype” in Methods). We measured the response according to the frequency changes of 16 partially overlapping T cell populations defined by the combined expression of the 4 AIMs used in the assay (**Figure 1A**, **Supplementary Table S3**, and Methods). Although hit identification based on the analysis of single populations, such as CD8^+^ T cells expressing CD137 in combination with IFNγ (CD137^+^ IFNγ^+^, **Figure 1B**) or alone (CD137^+^, **Figure 1C**) was sufficient to confidently detect reactivity to Peptivator pools or NIP random pools (RPs) in top responders, the evaluation of medium to low-level responses was more challenging. This was partly due to the level of the background signal, which, as shown for two of the top responders, HD 118 and HD114, was often high (**Figures 1D, E**; red and black rectangles). These data indicate that rarer but potentially relevant T cell specificities could have been overlooked in single population analysis of AIM markers due, among other reasons, to high and/or fluctuating background signal (noise).

### Reactivity score analysis of the T cell response

**Figure 2:**
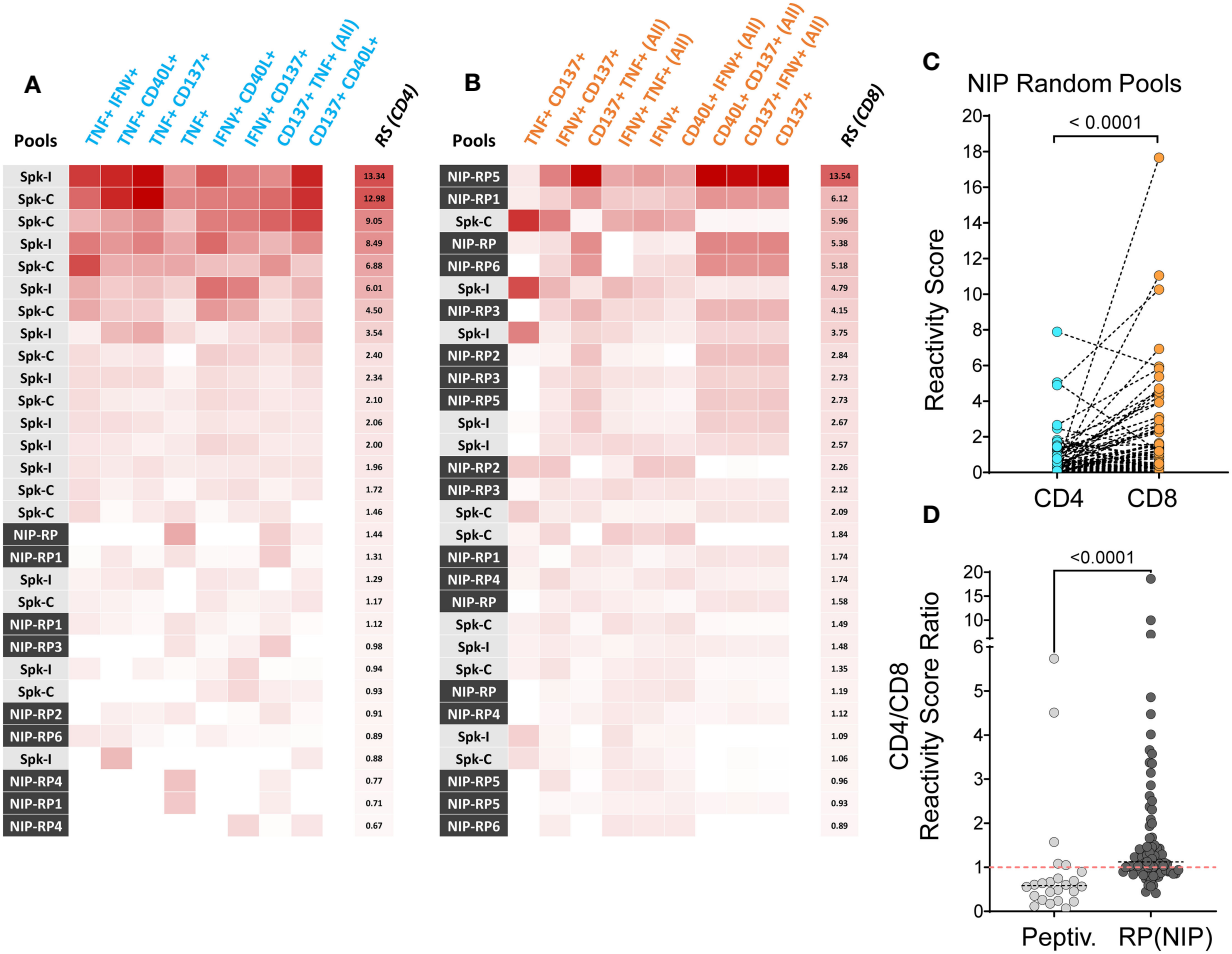
Reactivity score analysis of T cell response. **(A, B)** Top 30 responses to Peptivator pools (Spike-C or Spike-I) or NIP random pools (RP) according to the reactivity scores (RS) independently calculated for the CD4^+^ **(A)** and the CD8^+^ **(B)** T cell compartment. **(C)** CD4^+^ and CD8^+^ T cell reactivity scores following stimulation with NIP random pools (N = 90). **(D)** Comparison of CD8/CD4 RS ratios for the Peptivator pools (N = 24) and NIP random pools (N = 84). Mann-Whitney U test values are shown.

To minimize the dependence on the signal features of any specific marker and thus increase confidence in hit identification, we have calculated a reactivity score (RS) metric to determine the level of response in both CD8^+^ and CD4^+^ T cell subsets, using frequency data of selected cell populations defined by the combined expression of 4 AIMs, as previously described (4). We found that among 108 tests (PBMC from 12 donors challenged with 9 different stimuli), 21 of the top 30 RS for CD4^+^ T cells (70%) were obtained in response to Peptivator mixes (Spike-C or Spike-I; **Figure 2A**). Conversely, 18 of the top RS for CD8^+^ T cells (60%) were obtained following NIP RP stimulation (**Figure 2B**). Accordingly, the overall ranking of RSs associated to response to NIP RP was significantly higher in CD8^+^ T cells than in CD4^+^ T cells (**Figure 2C**; 0.46 vs. 0.30 median RS values; Wilcoxon signed-ranked test; P < 0.0001), and the CD8^+^/CD4^+^ T cell RS (CD8/CD4 RS) ratios after stimulation with NIP RP were significantly higher than the ratios measured after stimulation with the Peptivator mixes (**Figure 2D**; median value of 1.12 vs. 0.58; Mann-Whitney; P < 0.0001), a result consistent with the fact that differently than Spike-C and Spike-I pools, which are composed of 15 amino acid-long peptides and are thus expected to preferentially activate CD4^+^ T cells, NIP RPs are collections of shorter 9 to 10-mers peptide preferentially presented to CD8^+^ T cells by MHC class I molecules (18, 23, 24). These data not only support the validity of NIP predictions but also indicate that NIP peptides can be used for a more specific evaluation of vaccine induced CD8^+^ T cell response.

### Patterns identification and screening hit validation

Following a more granular examination of AIMs frequency data, we identified variation in response phenotypes and markers that were modulated together in some patients. The CD137 pattern (CD137 Pattern #1) in **Supplementary Figure S2** is shown as an example: Four partially overlapping T cell populations expressing CD137 alone or in combination with other AIMs clustered based on their frequency change following stimulation. Overall, we identified 7 activation patterns (**Supplementary Figure S3A**, **S3C**), 4 of which were associated with the CD8^+^ T cell response (**Supplementary Figure S3A**). A high degree of intra-donor and inter-donor heterogeneity in terms of response emerged from this analysis: For example, in some donors, such as HD 118, the response to stimulation (NIP RP A5) was primarily driven by the CD137 Pattern, whereas in others, such as HD 117 and HD 138, the response was associated to frequency upregulation in CD40L^+^ cell populations (CD40L Pattern; **Supplementary Figure S3B**). Overall, 9 of 12 donors showed detectable changes in at least one of the 7 activation patterns identified from the dataset (4 associated to CD8^+^ and 3 associated to CD4^+^ T cells; **Supplementary Figure S3**). We further compared the RS analysis results with the polyfunctional T cell responses of the donors by measuring the frequency of single, double, and triple positive events post-stimulation. A frequency threshold for positivity of 0.01% with at least 10 double positive events above background yielded fewer hits than the RS, even when the RS threshold was raised to 0.3 relative units (**Supplementary Figure S4A**). Because nearly all T cell responses were captured by IFNγ or TNF expression, our analysis focused on the frequencies of IFNγ^+^ and TNF^+^ cells that were single, double, and triple positive for TNF, IFNγ and/or the third most common marker, CD137 (**Supplementary Figure S4B**). Despite substantial heterogeneity in T cell response, the RS method showed higher sensitivity than the polyfunctional analysis and helped identify relevant NIP pools for further evaluation.

**Figure 3:**
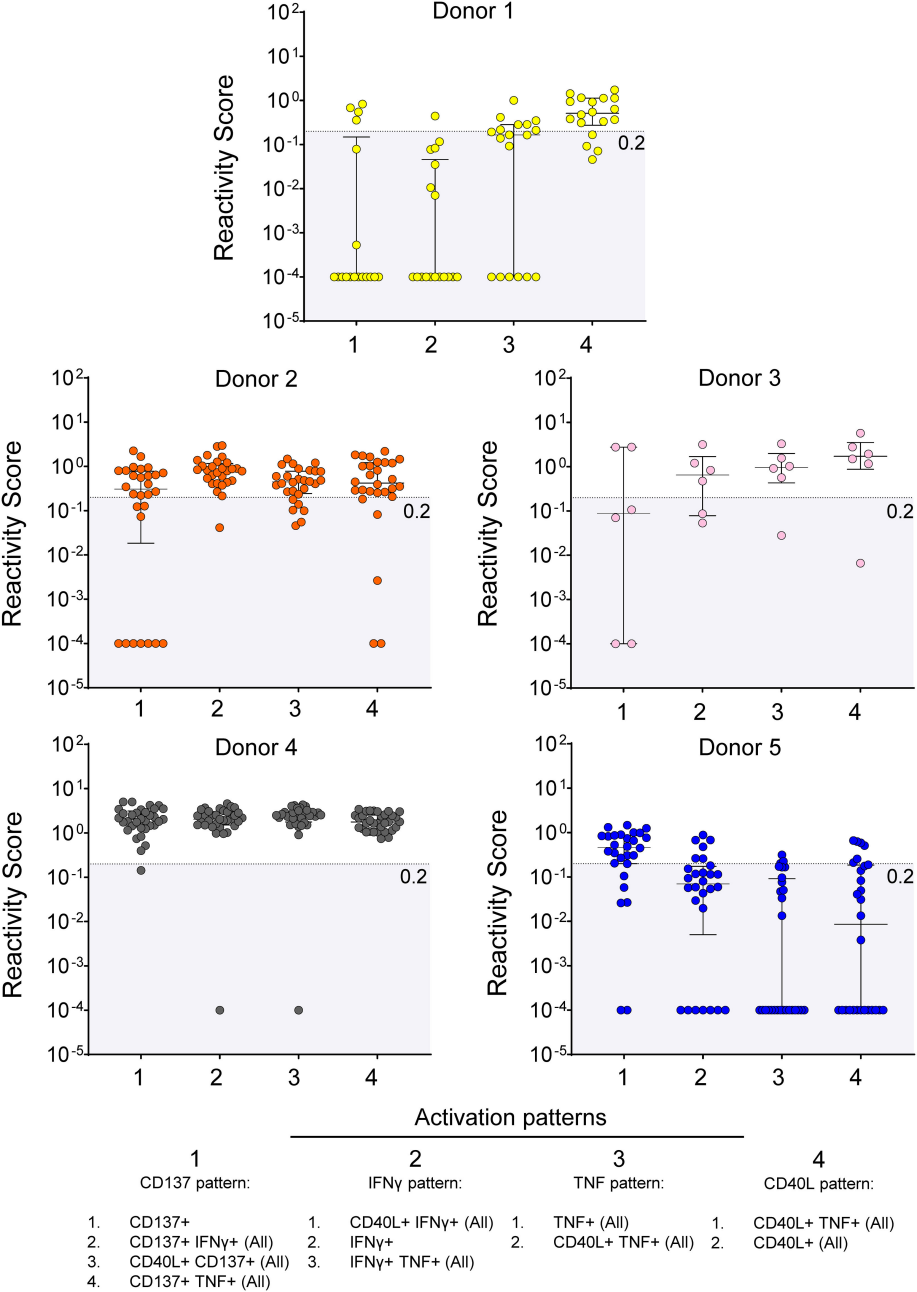
Screening hits validation. Response to stimulation with single peptides in 5 donors. The RS for each reactivity pattern is shown for each tested epitope. The empirical threshold for reactivity is shown (shaded area; RS ≤ 0.2). Donor 1 = HD 131; Donor 2 = HD 114; Donor 3 = HD 138; Donor 4 = HD 118; Donor 5 = HD 110.

Next, to validate the antigenicity at the single epitope level, we tested responses to individual peptides in CD8^+^ T cells from 5 of previously tested SARS-CoV-2-infected donors, who also received 3 doses of mRNA vaccines (**Figure 3**). By examining the change in the activation patterns, we found that each peptide elicited detectable response (RS > 0.2 units) in at least one of the 5 donors. In two donors (donor 2 and 3) reactivity was observed across all 4 patterns, while in others, peptides stimulation resulted in pattern-specific responses (see TNF, CD40L, or CD137 pattern-associated responses in donors 1 and 5). Notably, sequence analysis revealed that our screening independently identified epitopes with shared amino acid sequences (not shown).

**Figure 4:**
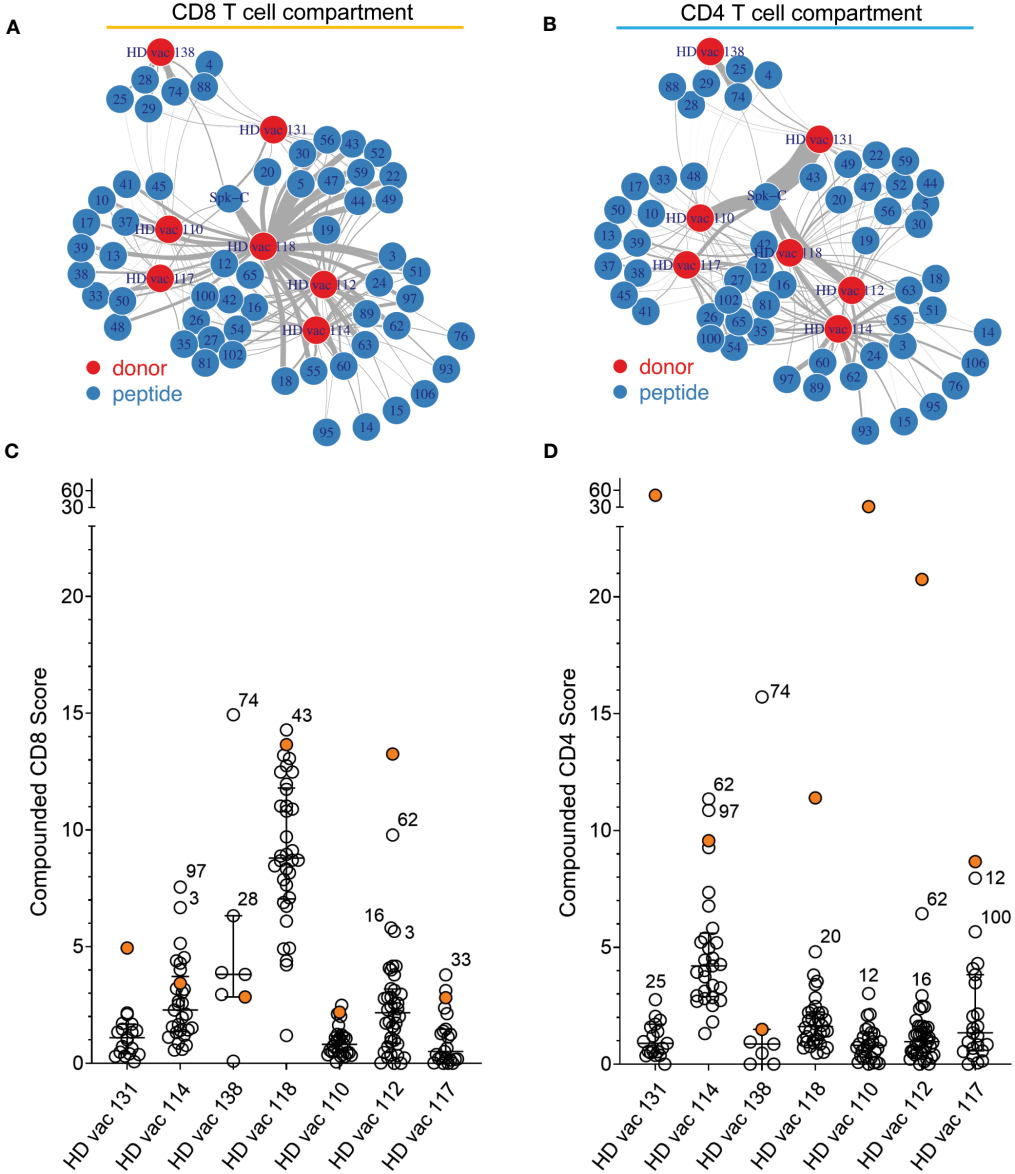
Peptide-donor network of compounded scores. **(A, B)** Peptide-donor network for the CD8^+^ **(A)** and the CD4^+^ **(B)** T cell compartment. Edge thickness is proportional to the magnitude of the compounded reactivity score for each of the 56 peptides (blue nodes). N = 7 donors (red nodes). **(C, D)** Comparison of the compounded reactivity scores of each peptide for **(A)** CD8^+^ and **(B)** CD4^+^ T cells. Orange dots represents the values for the Spike-C pool. Peptide ID is shown for top responses.

Networks depicting the relationship between donor (nodes; N = 7) and the magnitude of response (edges) to each peptide based on cumulative RS (Compounded Scores) calculated from the RS of each pattern (see Methods) confirmed that the NIP epitopes of length 9 and 10 amino acids prevalently activate CD8^+^ T cells (**Figure 4A**), whereas the Spike-C 15-mer-long peptide pool preferentially stimulates CD4^+^ T cells (**Figure 4B**). Patient comparison by compounded scores, showed that donor 4 (HD118) displayed a uniquely strong and broader CD8^+^ T cell response to NIP universal hotspots peptides (**Figures 4A, C**), and that only two donors (donor HD 131 and donor HD 112) responded more strongly to the Spike-C pool than any peptides (**Figure 4C**). Conversely, only 2 of 7 donors showed greater responses to NIP peptides than to the Spike-C pool in the CD4^+^ T cell compartment (donor HD 114 and donor HD 138; **Figure 4D**), confirming at the same time the reactivity bias previously observed with the NIP RPs and the robustness of NIP AI platform predictions.

### Epitope breakdown by region and identification of new antigenic determinants

**Figure 5:**
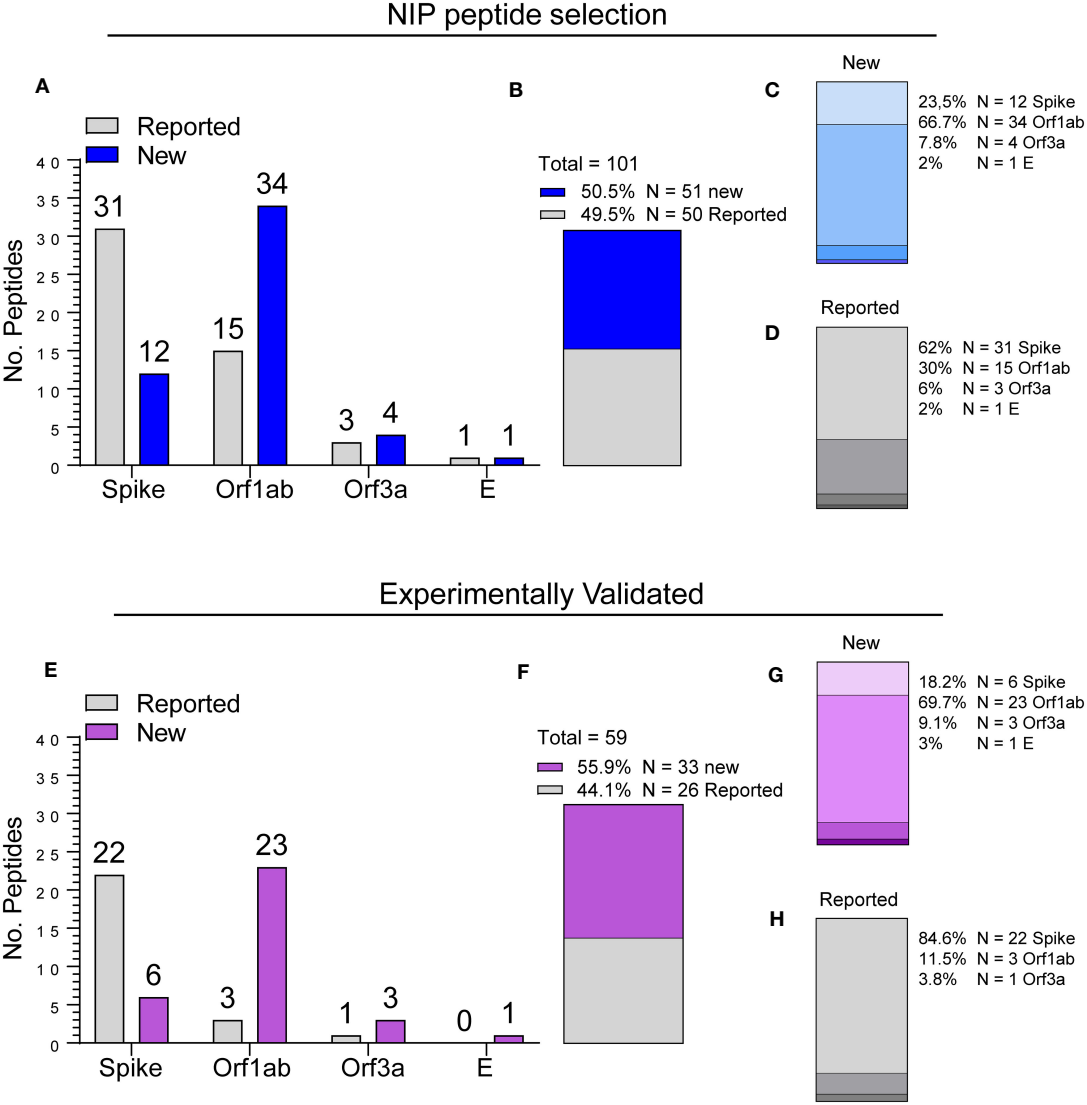
Epitope breakdown by region and identification of new antigenic determinants. **(A)** Number of NIP peptides by location (Spike, Orf1ab, Orf3a, E) and novelty status (Reported vs. New). **(B)** Breakdown by novelty status. **(C, D)** Breakdown by location of new **(C)** and already reported **(D)** peptides as shown in **(B)**. **(E)** Number of experimentally validated NIP peptides organized by location and novelty status as shown in **(A)**. **(F-H)** Breakdown by novelty status and location as shown in **(B, D)**.

Epitope breakdown by region of origin and novelty status according to curated scientific literature data retrieved on May 5^th^, 2023, from The Immune Epitope Database (IEDB) (11), indicated that while 49.5% of the NIP peptides originally selected for the screening (N = 101) have already been described, 50.5% were never reported before (**Figures 5A, B**). Moreover, we found that the majority of the newly found epitopes (67%) originated from the Orf1ab region (**Figure 5C**), whereas 62% of already reported epitopes originated from the S protein (**Figure 5D**). A similar pattern was observed in the 59 validated NIP peptides selected for the NIP pool (**Figure 5E**, **Supplementary Table S2**) among which 33/59 (56%) were never reported before (**Figure 5F**). Of note, among the newly identified epitopes, Orf1ab region peptides were overrepresented (23/33, 70%; **Figure 5G**), while enrichment in S protein peptides was seen among epitopes that were already been reported (22/26, 85%; **Figure 5H**). These results indicate that the universal hotspots detected by the NIP algorithm not only harbour known immunogenic epitopes, but also contain novel antigenic determinants of potential interest.

### NIP pool performance testing

**Figure 6:**
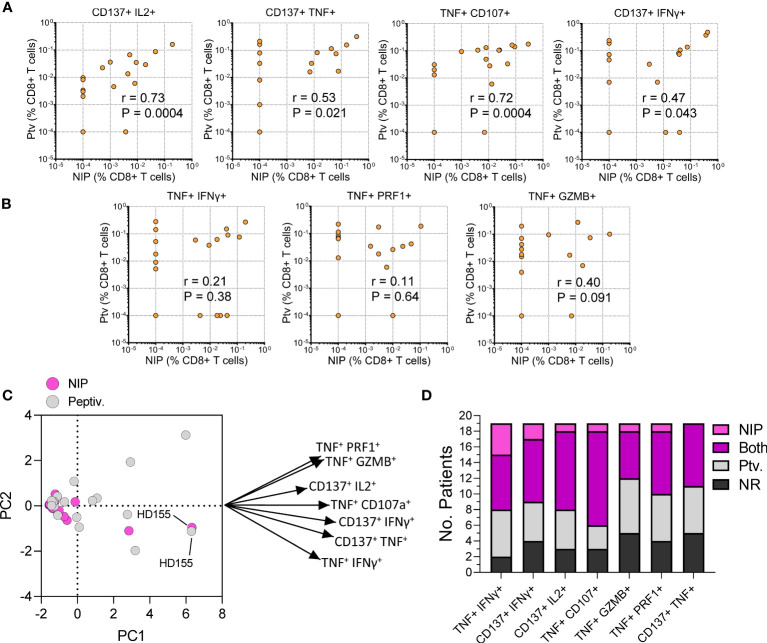
Performance testing of the Spike NIP pool. **(A, B)** Comparison of CD8^+^ T cell response to Peptivator mix (Ptv; y-axis) and Spike NIP pool (NIP; x-axis) in 19 vaccinated healthy donors. T cell populations are shown above each graph. Spearman’s r and P values are shown. **(C)** PCA analysis and Loading plot dimensions for Spike NIP pool and Peptivator mix treatments. **(D)** Breakdown of the response by T cell population and stimulus type: NIP, Spike NIP pool; Ptv., Peptivator mix (Spike-I + Spike-C); NR, No Response.

Having validated the immunogenicity of single NIP peptides, we pooled the top S protein NIP peptides and used the resulting mix (Spike NIP pool) to test CD8^+^ T cell response in 19 healthy double-vaccinated donors using a modified stimulation protocol and an extended flow panel (see Methods). We found that the response to the Spike NIP pool and Peptivator mix (Spike-C + Spike-I) were comparable (**Figure 6**). Specifically, there was a significant correlation between the level of response attained by stimulation with the NIP pool and the Peptivator mix in different T cell populations defined by the co-expression of multiple AIMs including CD137^+^ IL2^+^ (Spearman’s r = 0.73; P = 0.0004), CD137^+^ TNF^+^ (Spearman’s r = 0.53; P = 0.021), TNF^+^ CD107^+^ (Spearman’s r = 0.72; P = 0.0004), and CD137^+^ IFNγ^+^ (Spearman’s r = 0.47; P = 0.043) T cells (**Figure 6A**). Although the correlation was lost when response was evaluated by looking at other CD8^+^ T cell populations, such as TNF^+^ IFNγ^+^ (Spearman’s r = 0.21; P = 0.38), TNF^+^ PRF1^+^ (Spearman’s r = 0.11; P = 0.64) or TNF^+^ GZMB^+^ (Spearman’s r = 0.40; P = 0.091) cells (**Figure 6B**), PCA analysis of all the markers confirmed a substantial similarity in the response to the two stimuli in terms of immunological polyfunctionality (**Figure 6C**). Notably, we observed that when assessing T cell populations defined by specific combinations of markers, such as TNF and IFNγ, or CD137 and IFNγ, vaccine-induced reactivity for some donors could only be detected after stimulation with the Spike NIP pool. Overall, 7 donors displayed response frequencies < 0.01%, independently of the combination of AIM markers examined or the stimulus type, whereas 63% (12/19) of the donors showed CD8^+^ T cell reactivity toward the Spike NIP pool. These data validate our analysis and demonstrate the utility of the Spike NIP pool as a complementary screening tool for the assessment of vaccine-induced T cell response, particularly in assays based on the use of a limited number of markers.

## Discussion

Here, we report the experimental validation of SARS-CoV-2 T cell epitopes previously identified by an AI prediction algorithm as being immunogenic target candidates for a potential COVID-19 T cell-based universal vaccine (1). All epitopes validated in this study were derived from immunogenic viral hotspots predicted to be immunodominant in the human population and were selected according to their predicted affinity for prevalent HLAs in the Norwegian population. In addition to validating these epitopes as a complementary screening tool for the assessment of vaccine-induced T cell reactivity, we have identified novel antigenic peptides from both the S protein and the non-spike regions of the virus.

Many T cell epitope prediction tools rely on HLA binding affinity as a proxy for T cell immunogenicity (25–30). More recent tools use mass spectrometry immunopeptidome data to perform prediction of immunogenic T cell epitopes for MHC-I (*e.g.*, the proprietary model known as EDGE) (31) and/or MHC-II alleles (*e.g.*, the MARIA models) (32). These and other models are similar to NIP and are an important advance, as they are based on cell surface-presented antigens using mass spec immunopeptidome data as a source of training data (31, 33–38). However, this approach performs well for only a small minority of HLA alleles in the human population, because sufficient binding affinity training data exists only for this small minority (11, 39) and HLA binding affinity alone is a necessary but not sufficient property to define true immunogenicity. Furthermore, although some of these tools use flanking sequences to capture some elements of antigen processing, many algorithms rely on mono HLA-allelic cell lines to train their models, and therefore miss the endogenous antigen processing and presentation features emerging from the competition among several HLA alleles naturally present in multiallelic cells. In addition to this artefact, many of these tools have trained their algorithms to predict, in an improved manner, aspects of HLA-peptide binding from the mass spec immunopeptidome data but not HLA-peptide cell surface presentation or T cell immunogenicity. To overcome these limitations, NIP utilizes an ensemble machine-learning model that is trained on a broad set of both publicly available and proprietary mass spec immunopeptidome data, and combines four dedicated HLA binding affinity models, 13 antigen processing models, and other protein and expression features to predict the ability of the peptide to navigate through the endogenous antigen processing and presentation (AP) machinery of the cell and be ultimately presented on the cell membrane as an HLA:peptide complex. Each model learns the interplay between the other models in a holistic manner capturing the true determinants of antigen presentation to the cell surface and T cell immunogenicity, above and beyond HLA binding characteristics alone. Candidate peptides with high AP potential are also probed using additional bioinformatics toolkits to quantify the degree of “foreignness” relative to the human proteome and therefore their likelihood to activate T cell response in a patient.

Hits from the *ex vivo* validation screening were identified based on the analysis of T cell populations expressing CD137, TNF, IFNγ, or CD40L. These four AIMs are typically used alone or in combination to evaluate T cell reactivity (2, 3, 40). We observed remarkable interpatient heterogeneity in terms of the immunophenotype of the vaccine-specific T cell response. For example, T cell activation in some donors was primarily marked by CD137 upregulation, but in others, changes in CD40L expression were the predominant response. Furthermore, we found that NIP peptide pools significantly outperformed Peptivator megapools in terms of identifying patients with positive anti-SARS-CoV-2 CD8^+^ T cells responses, even though response was detected in CD4^+^ T cells from most donors according to changes in at least one of the 3 activation patterns defined in the study. Nonetheless, CD4^+^ T cell and CD8^+^ T cell responses were generally independent except for a few donors who displayed a particularly strong response to the vaccine. These results may be partly due to the different length of the peptides used in the study as well as on the likelihood that undetected responses may fall below the quantification limit of the assay. In this regard, it is worth noting that 15-mers peptides not only can directly bind MHC Class II molecules and be presented to CD4 T cells, but they can also be processed to 8 to 12 amino acid-long peptides capable of binding Class I and being presented to CD8 T cells.

Given the significant inter-donor heterogeneity, we sought to develop a data analysis strategy based on the combination of the frequency values of T cell populations defined by the expression of all possible AIMs combinations, as shown in **Figure 1A**, to compute a global reactivity score for each T cell compartment (CD8^+^ and CD4^+^ T cells). A similar approach was previously used to investigate vaccine-induced T cell response in a cohort of immunocompromised bone marrow-transplanted patients (4). This data-driven, heuristic method improved detection sensitivity and helped identify additional immunogenic sequences that would have been otherwise missed. Furthermore, we observed that while the set of minimal NIP-derived 9 to 10-mers tested in this study preferentially activated CD8^+^ T cells, the Peptivator mix pool, which is composed of 15-mers peptides, preferentially stimulated CD4^+^ T cells. These results not only corroborated the notion that peptide length is an important factor in determining the type of T cell response (18, 23, 41–45), but also provided further cross validation for the analytical methodology used in the study. While these findings provide a promising avenue for future research, the study is limited by the sample number and nationality of patient samples. The inclusion of diverse demographic groups, multiple geographical locations, and different stages of infection or vaccination status will provide more robust and generalizable data to further validate the predictive capabilities of AI platforms such as NIP, and the effectiveness of these peptides as screening tools.

Since January 11^th^, 2020, when the first death from COVID-19 was reported by the Chinese government, SARS−CoV−2 has infected nearly 800 million individuals (46) and demonstrated a remarkable capability of evolving, particularly through mutations within the S protein (19). By interrogating the Immune Epitope Database and Analysis Resource (IEDB), a repository of and reference resource for epitopes associated to infection, autoimmunity, and allergic processes (47), we found that most of the new peptides validated in the study belonged to non-spike regions, including the Orf1ab region, the accessory protein Orf3a, or the envelope (E) protein. While the Orf1ab encodes two non-structural polyproteins, pp1a and pp1ab, which are directly transcribed by cellular ribosomes and proteolytically processed into 16 non-structural proteins (48), the Orf3a may act as a non-selective permeable cation channel with high sequence homology to the SARS CoV-1 viroporin, and is likely involved in various steps of the life cycle of the virus including endocytosis, lysosomal dynamics, viral genome transcription, and virion exocytosis (49, 50). Furthermore, it has been postulated that specific mutations within these regions may have been positively selected via epistatic interactions early in the pandemic (51). Indeed, in SARS-CoV-2, the Orf3a protein may work in coordination with the E protein, another highly conserved essential structural protein with viroporin function (52), to help release the virion from the host cell through the modification of the membrane permeability (53). The identification of novel antigenic peptides from non-spike regions is a result of interest because most of the studies on SARS-CoV-2 evolvability and antigenicity have been focusing on S protein (19, 20).

The NIP AI platform has been previously used to demonstrate the high level of conservation of predicted T cell epitopes among all mutated peptides identified in variants of concern (VOCs) (54). However, additional studies will be necessary to establish the functional importance and the hypothetical evolutionary stability of these regions, particularly, to determine whether megapools composed of non-structural peptides could be used as a screening tool for the detection of virus-specific T cell response toward VOCs carrying divergent S or N protein sequences, which are expected to emerge. Moreover, future research should focus on examining the extent of cross-reactivity of these peptides with other coronaviruses, to inform the development of broader spectrum vaccines or therapies. Finally, considering that some immunocompromised patients who fail to generate protective antibody levels still retain a cell-mediated immune response (6, 55–57), these peptides could be employed as a complementary screening tool for the assessment of vaccine-generated CD8^+^ T cell immune response elicited by vaccines in high-risk patients with a compromised immune system.

## Data availability statement

The original contributions presented in the study are included in the article/**Supplementary Material**, further inquiries can be directed to the corresponding authors.

## Ethics statement

The studies involving humans were approved by Health Region South-East Regional Ethics committee. The studies were conducted in accordance with the local legislation and institutional requirements. The participants provided their written informed consent to participate in this study.

## Author contributions

LF: Conceptualization, Data curation, Formal Analysis, Investigation, Methodology, Supervision, Validation, Visualization, Writing – original draft, Writing – review and editing. BM: Data curation, Formal Analysis, Methodology, Writing – review and editing. ST: Data curation, Formal Analysis, Methodology, Writing – review and editing. VC: Data curation, Methodology, Writing – review and editing. JO: Data curation, Methodology, Writing – review and editing. MG: Data curation, Methodology, Writing – review and editing. ES: Formal Analysis, Visualization, Writing – review and editing. HK: Formal Analysis, Writing – review and editing. RA: Formal Analysis, Visualization, Writing – review and editing. VG: Formal Analysis, Writing – review and editing, Supervision, Visualization. RS: Data curation, Formal Analysis, Funding acquisition, Methodology, Resources, Writing – review and editing. TC: Data curation, Funding acquisition, Resources, Writing – review and editing, Formal Analysis, Methodology. LM: Conceptualization, Data curation, Funding acquisition, Investigation, Project administration, Resources, Supervision, Writing – original draft, Writing – review and editing.
