# Corrigendum: Experimental validation of immunogenic SARS-CoV-2 T cell epitopes identified by artificial intelligence

**DOI:** 10.3389/fimmu.2024.1377041

**Published:** 2024-02-21

**Authors:** Lorenzo Federico, Brandon Malone, Simen Tennøe, Viktoriia Chaban, Julie Røkke Osen, Murat Gainullin, Eva Smorodina, Hassen Kared, Rahmad Akbar, Victor Greiff, Richard Stratford, Trevor Clancy, Ludvig Andre Munthe

**Affiliations:** ^1^ Department of Immunology, Oslo University Hospital, Oslo, Norway; ^2^ KG Jebsen Centre for B cell Malignancies, Institute of Clinical Medicine, University of Oslo, Oslo, Norway; ^3^ NEC OncoImmunity AS, Oslo, Norway; ^4^ Institute of Clinical Medicine, Oslo University Hospital, Oslo, Norway

**Keywords:** T cell, COVID-19, CD8+ lymphocytes, CD4+ lymphocytes, vaccine, SARS-CoV-2, artificial intelligence, antigen (Ag)

## Text correction

In the published article, there were two errors: The “Material and methods” section preceded the “Introduction” section, and there was an inaccuracy in the wording of a sentence in the “Reagents” sub-section of “Material and methods”.

The “Introduction” now precedes the “Material and methods”, and a correction has been made to a sentence in “Materials and methods”, sub-section “Reagents”.

This sentence previously stated:

“Peptides were synthesized by GenScript (Piscataway, NJ, USA) at a purity ≥85%, and used at a final concentration of 1.5mg/mL each.”

The corrected sentence appears below:

“Peptides were synthesized by GenScript (Piscataway, NJ, USA) at a purity ≥85% and stored at a final concentration of 1.5mg/mL.”

The authors apologize for these errors and state that this does not change the scientific conclusions of the article in any way. The original article has been updated.

