# Supplementary material for: Experimental validation of immunogenic SARS-CoV-2 T cell epitopes identified by artificial intelligence
Source: Front Immunol. 2023 Nov 17;14:1265044. doi: 10.3389/fimmu.2023.1265044 (PMC10691274; doi:10.3389/fimmu.2023.1265044)
Supplement: Supplementary file 1 [file Presentation_1.pdf]

Supplementary Material for:

Experimental validation of immunogenic SARS-CoV-2 T cell epitopes identified by artificial intelligence.

Lorenzo Federico[1,2*], Brandon Malone[3], Simen Tennøe[3], Viktoriia Chaban[1,2], Julie Røkke Osen[1,2], Murat Gainullin[1,2], Eva Smorodina[1,4], Hassen Kared[1,2], Rahmad Akbar[1,4], Victor Greiff[1,4], Richard Stratford[3], Trevor Clancy[3], and Ludvig Andre Munthe[1,2*]

Author affiliations:
[1] Department of Immunology, Oslo University Hospital, Oslo, Norway
[2] KG Jebsen Centre for B cell Malignancies, Institute of Clinical Medicine, University of Oslo, Norway
[3] NEC OncoImmunity AS, Oslo, Norway
[4] Institute of Clinical Medicine, Oslo University Hospital, Oslo, Norway

***Corresponding authors:**

Lorenzo Federico (lorenzo.federico@medisin.uio.no) and Ludvig Andre Munthe (l.a.munthe@medisin.uio.no)

Supplementary Material Summary:

- **Supplementary Figure S1.**
- **Supplementary Figure S2.**
- **Supplementary Figure S3.**
- **Supplementary Table S3**

Supplementary Figure S1. Assay set-up and flow data gating strategy. Set-up of the screening plate. PBMC from multiple donors (rows) were stimulated with Spike-I (Spk-I), Spike-C (Spk-C), or NIP random pools (RP1... RP_x), as indicated. NS = non-stimulated control (signal background). The gating strategy is shown.

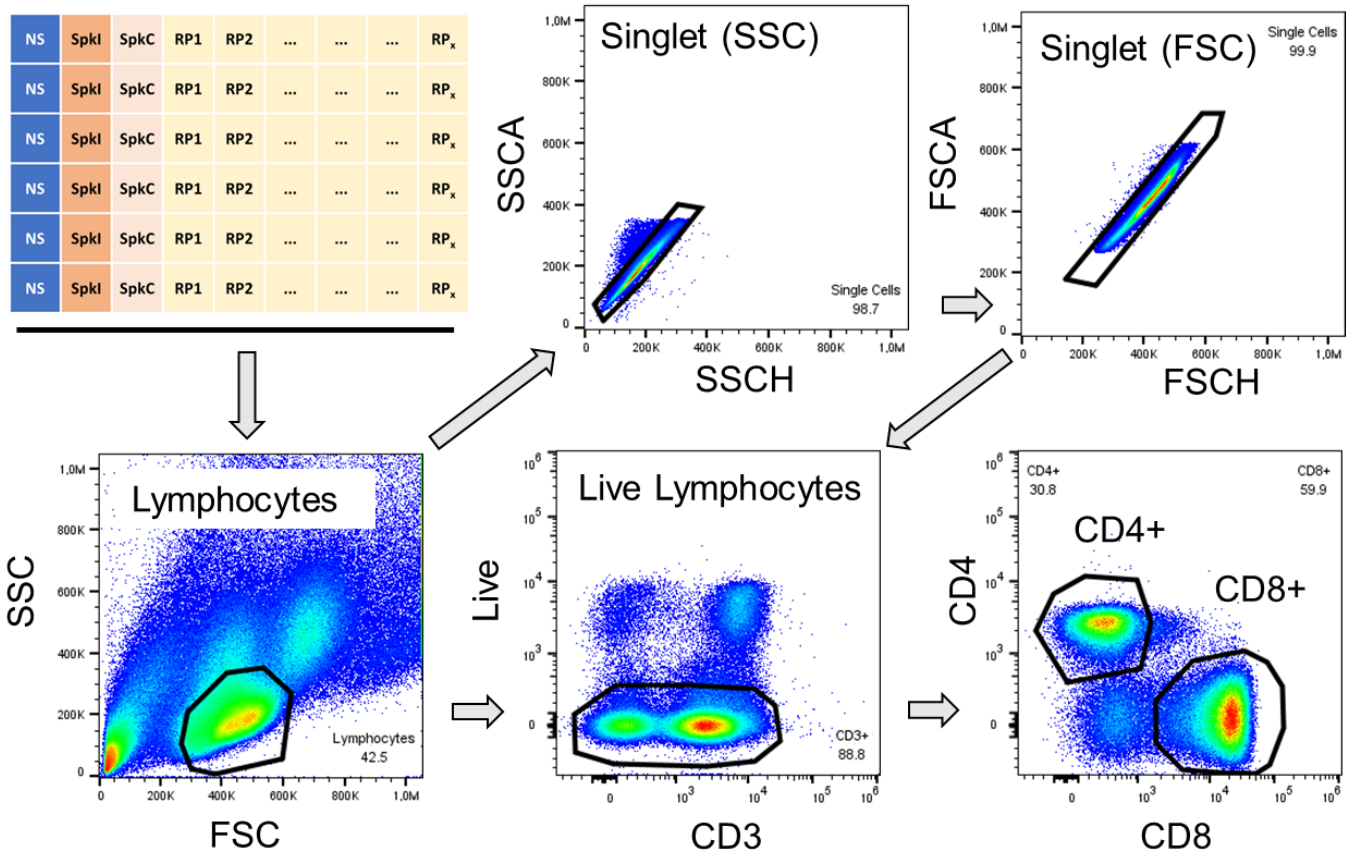

Supplementary Figure S2. **Activation pattern identification.** A heatmap of the four subpopulations that constitute the CD137 pattern is shown. Population frequencies are shown for each patient (columns) and treatment (Spike-C, Spike-I, and NIP RP A1-7).

Avg., % = Frequency average of the four co-modulated T cell subpopulations of the CD137 Pattern. RP = Random Pool.

		HD104	HD110	HD112	HD114	HD117	HD118	HD121	HD124 t2	HD131	HD132	HD136	HD138
**CD137+**	**Spike-C**	0,06	-0,10	-0,56	0,61	0,22	0,33	-1,30	0,29	0,95	-0,08	0,18	-1,15
**CD137+ IFNG+ All**		0,17	-0,10	-0,61	0,60	0,20	0,38	-1,21	0,30	0,96	-0,03	0,19	-1,01
**CD137+ TNF+ All**		0,18	-0,15	-0,49	0,60	0,26	0,36	-1,14	0,26	0,98	-0,14	0,31	-1,16
**CD40L+ CD137+ All**		-0,02	-0,26	-0,57	0,50	0,20	0,33	-1,18	0,27	0,97	-0,18	0,25	-0,93
**CD137 Pattern**	**Avg., % >>>>**	**0,10**	**-0,15**	**-0,56**	**0,58**	**0,22**	**0,35**	**-1,21**	**0,28**	**0,97**	**-0,11**	**0,23**	**-1,06**
**CD137+**	**Spike-I**	-0,46	0,61	-0,53	0,36	1,69	0,83	-1,79	1,20	1,42	0,17	0,24	-0,22
**CD137+ IFNG+ All**		-0,44	0,63	-0,51	0,33	1,69	0,87	-1,81	1,29	1,51	0,23	0,25	-0,11
**CD137+ TNF+ All**		-0,45	0,58	-0,53	0,40	1,79	0,99	-1,66	1,22	1,57	0,15	0,32	-0,19
**CD40L+ CD137+ All**		-0,50	0,65	-0,54	0,30	1,69	0,73	-1,81	1,16	1,43	0,19	0,20	-0,13
**CD137 Pattern**	**Avg., % >>>>**	**-0,46**	**0,62**	**-0,53**	**0,35**	**1,72**	**0,86**	**-1,77**	**1,22**	**1,48**	**0,18**	**0,25**	**-0,16**
**CD137+**	**RP-A7**	-0,10	0,92	0,27	-0,92	-0,37	4,18	-1,20	0,22	-0,12	-0,08	-0,04	-1,15
**CD137+ IFNG+ All**		0,00	0,97	0,39	-0,93	-0,42	4,25	-1,25	0,22	-0,06	-0,11	-0,03	-1,12
**CD137+ TNF+ All**		-0,06	0,93	0,31	-0,92	-0,27	4,03	-1,23	0,20	-0,13	-0,11	0,00	-1,19
**CD40L+ CD137+ All**		-0,16	0,89	0,31	-0,95	-0,28	4,12	-1,19	0,27	-0,14	-0,16	-0,10	-1,00
**CD137 Pattern**	**Avg., % >>>>**	**-0,08**	**0,93**	**0,32**	**-0,93**	**-0,34**	**4,15**	**-1,22**	**0,23**	**-0,11**	**-0,11**	**-0,04**	**-1,12**
**CD137+**	**RP-A6**	-0,95	-0,54	-0,58	-1,56	-0,80	3,76	-2,43	-0,11	-0,21	0,12	-0,11	-2,18
**CD137+ IFNG+ All**		-0,92	-0,57	-0,49	-1,52	-0,68	3,84	-2,41	-0,11	-0,15	0,16	-0,09	-2,16
**CD137+ TNF+ All**		-0,92	-0,58	-0,49	-1,52	-0,68	3,64	-2,29	-0,11	-0,26	0,11	-0,13	-2,19
**CD40L+ CD137+ All**		-1,03	-0,67	-0,56	-1,61	-0,75	3,84	-2,43	-0,12	-0,05	0,00	-0,11	-2,09
**CD137 Pattern**	**Avg., % >>>>**	**-0,96**	**-0,59**	**-0,53**	**-1,55**	**-0,73**	**3,77**	**-2,39**	**-0,11**	**-0,17**	**0,10**	**-0,11**	**-2,16**
**CD137+**	**RP-A5**	-1,09	0,25	1,92	0,06	-0,50	8,67	-1,81	0,35	-0,37	-0,15	-0,19	-2,29
**CD137+ IFNG+ All**		-1,08	0,30	1,88	0,12	-0,51	8,80	-1,81	0,40	-0,23	-0,17	-0,17	-2,09
**CD137+ TNF+ All**		-1,02	0,33	1,93	0,10	-0,41	8,78	-1,91	0,33	-0,36	-0,24	-0,14	-2,21
**CD40L+ CD137+ All**		-1,19	0,25	1,86	-0,01	-0,48	8,64	-1,65	0,37	-0,25	-0,16	-0,21	-2,20
**CD137 Pattern**	**Avg., % >>>>**	**-1,10**	**0,28**	**1,90**	**0,07**	**-0,48**	**8,72**	**-1,80**	**0,36**	**-0,30**	**-0,18**	**-0,17**	**-2,20**
**CD137+**	**RP-A4**	-0,31	0,46	-0,38	-0,19	0,44	0,50	-1,64	-0,12	0,12	-0,05	-0,03	-1,27
**CD137+ IFNG+ All**		-0,34	0,56	-0,32	-0,18	0,43	0,56	-1,68	-0,12	0,20	0,01	-0,02	-1,23
**CD137+ TNF+ All**		-0,33	0,50	-0,36	-0,17	0,47	0,60	-1,64	-0,08	0,03	-0,08	-0,06	-1,34
**CD40L+ CD137+ All**		-0,41	0,35	-0,38	-0,21	0,56	0,58	-1,64	-0,18	0,12	-0,18	-0,08	-1,18
**CD137 Pattern**	**Avg., % >>>>**	**-0,35**	**0,47**	**-0,36**	**-0,18**	**0,47**	**0,56**	**-1,65**	**-0,12**	**0,12**	**-0,07**	**-0,05**	**-1,26**
**CD137+**	**RP-A3**	-0,74	0,17	1,56	2,56	0,76	-2,36	-1,23	-0,10	-0,13	-0,08	-0,27	-1,83
**CD137+ IFNG+ All**		-0,73	0,14	1,53	2,62	0,84	-2,11	-1,24	-0,04	-0,05	-0,13	-0,27	-1,73
**CD137+ TNF+ All**		-0,74	0,15	1,54	2,59	0,92	-2,37	-1,14	-0,13	-0,20	-0,11	-0,26	-1,82
**CD40L+ CD137+ All**		-0,88	0,14	1,54	2,47	0,80	-2,37	-1,20	-0,16	-0,05	-0,19	-0,30	-1,80
**CD137 Pattern**	**Avg., % >>>>**	**-0,77**	**0,15**	**1,54**	**2,56**	**0,83**	**-2,30**	**-1,20**	**-0,11**	**-0,11**	**-0,13**	**-0,28**	**-1,80**
**CD137+**	**RP-A2**	-0,74	-0,13	-0,19	2,18	-0,77	-0,01	-1,68	0,04	-0,28	0,05	-0,17	-1,76
**CD137+ IFNG+ All**		-0,76	-0,09	-0,20	2,21	-0,70	0,16	-1,75	0,03	-0,26	0,05	-0,22	-1,72
**CD137+ TNF+ All**		-0,73	0,00	-0,16	2,18	-0,62	-0,10	-1,61	0,09	-0,28	-0,02	-0,24	-1,74
**CD40L+ CD137+ All**		-0,82	-0,24	-0,15	2,11	-0,78	0,02	-1,65	0,02	-0,22	-0,02	-0,20	-1,71
**CD137 Pattern**	**Avg., % >>>>**	**-0,76**	**-0,12**	**-0,17**	**2,17**	**-0,72**	**0,02**	**-1,67**	**0,05**	**-0,26**	**0,02**	**-0,21**	**-1,73**
**CD137+**	**RP-A1**	-0,29	-0,07	0,93	-0,47	0,06	0,06	-1,69	0,04	-0,52	0,09	-0,07	3,52
**CD137+ IFNG+ All**		-0,31	-0,11	0,93	-0,46	0,07	0,16	-1,67	0,01	-0,42	0,11	-0,05	3,71
**CD137+ TNF+ All**		-0,22	-0,07	1,04	-0,45	0,08	0,02	-1,59	0,15	-0,47	0,06	-0,05	3,58
**CD40L+ CD137+ All**		-0,32	-0,08	0,98	-0,51	0,12	0,01	-1,74	0,02	-0,49	0,07	-0,13	3,67
**CD137 Pattern**	**Avg., % >>>>**	**-0,28**	**-0,08**	**0,97**	**-0,47**	**0,08**	**0,06**	**-1,67**	**0,06**	**-0,47**	**0,08**	**-0,08**	**3,62**

Supplementary Figure S3. Immunophenotype heterogeneity of the T cell response to antigenic challenge. (A) Heatmaps of the reactivity patterns for the $CD8^+$ T cell subset following stimulation with Peptivator mixes (Spk-C or Spk-I) or NIP RPs (A1-A7). Donor's ID is shown (top row; N = 12). (B) Interpatient variability of the activation patterns for the $CD8^+$ T cell subset following stimulation with NIP RP A5. (C) Heatmaps of the reactivity patterns for the $CD4^+$ T cell subset.

A

1 - CD137 PATTERN
CD137+
CD137+ IFNγ+ (All)
CD40L+ CD137+ (All)
CD137+ TNF+ (All)

Scale: 0–20

	104	110	112	114	117	118	121	131	132	136	138	124 t2
A1	0,00	0,00	2,37	0,00	0,20	0,15	0,00	0,00	0,20	0,00	8,87	0,14
A2	0,00	0,00	0,00	5,31	0,00	0,11	0,00	0,00	0,06	0,00	0,00	0,11
A3	0,00	0,37	3,79	6,26	2,03	0,00	0,00	0,00	0,00	0,00	0,00	0,00
A4	0,00	1,14	0,00	0,00	1,17	1,37	0,00	0,29	0,01	0,00	0,00	0,00
A5	0,00	0,69	4,65	0,16	0,00	21,38	0,00	0,00	0,00	0,00	0,00	0,89
A6	0,00	0,00	0,00	0,00	0,00	9,24	0,00	0,00	0,23	0,00	0,00	0,00
A7	0,00	2,27	0,78	0,00	0,00	10,16	0,00	0,00	0,00	0,00	0,00	0,56
Spk.C	0,25	0,00	0,00	1,42	0,54	0,86	0,00	2,37	0,00	0,57	0,00	0,68
Spk.I	0,00	1,51	0,00	0,85	4,20	2,10	0,00	3,64	0,45	0,62	0,00	2,98

2 - IFNG PATTERN
CD40L+ IFNG+ (All)
IFNγ+ TNF+(All)
IFNγ+

Scale: 0–20

	104	110	112	114	117	118	121	131	132	136	138	124 t2
A1	0,00	0,00	1,40	0,29	0,18	0,10	0,26	0,57	0,10	0,00	4,65	0,34
A2	0,00	0,21	1,24	0,80	0,43	3,59	0,00	0,40	0,03	0,00	0,22	0,20
A3	0,00	0,01	2,23	2,89	2,67	1,29	0,84	0,86	0,03	0,00	1,31	0,50
A4	0,00	1,55	1,82	0,50	0,28	1,98	0,10	0,39	0,51	0,00	0,49	0,23
A5	0,00	1,22	1,32	1,79	0,00	7,79	1,54	1,15	0,00	0,01	1,32	1,38
A6	0,00	0,00	2,11	0,80	0,90	2,02	0,56	0,91	0,43	0,02	0,16	0,25
A7	0,66	1,13	2,16	0,00	0,11	1,10	0,13	0,20	0,00	0,43	1,01	0,31
Spk.C	1,50	0,14	0,09	0,25	0,86	7,34	4,03	1,10	0,36	2,39	1,86	1,83
Spk.I	0,02	1,36	0,72	0,04	1,52	4,43	1,56	2,22	0,96	0,73	1,40	2,85

3 - TNF PATTERN
TNF+
CD40L+ TNF+ (All)

Scale: 0–20

	104	110	112	114	117	118	121	131	132	136	138	124 t2
A1	0,69	0,00	3,60	0,22	0,54	0,00	2,95	0,00	0,00	0,00	2,49	2,31
A2	0,00	0,86	1,22	0,23	1,16	0,24	1,89	0,62	0,00	0,00	1,16	1,12
A3	0,00	0,38	0,27	0,34	3,31	0,00	1,92	0,24	0,18	0,00	1,24	0,00
A4	0,00	0,42	0,67	0,53	1,39	2,37	1,06	0,00	0,00	0,00	0,83	0,40
A5	0,30	1,97	0,31	0,15	0,83	2,55	2,16	0,41	0,00	0,31	2,09	0,77
A6	0,00	0,00	1,01	0,32	1,35	0,00	2,73	0,81	0,03	0,00	0,76	0,19
A7	0,30	0,21	0,55	0,00	2,40	0,00	0,77	0,00	0,02	0,14	1,56	0,79
Spk.C	2,58	0,00	0,76	0,21	0,84	6,92	4,51	1,64	0,00	3,56	1,90	0,22
Spk.I	0,00	0,26	0,06	0,37	1,31	7,82	4,32	5,51	0,20	0,64	0,75	1,00

4 - CD40L PATTERN
CD40L+ TNF+ (All)
CD40L+

Scale: 0–20

	104	110	112	114	117	118	121	131	132	136	138	124 t2
A1	0,13	0,00	3,14	0,00	1,62	0,00	1,16	0,43	0,00	0,00	4,69	1,06
A2	0,00	0,00	1,46	0,00	0,41	1,01	1,52	1,69	0,00	0,00	1,59	0,49
A3	0,00	0,24	0,00	0,00	2,41	0,00	1,52	1,59	0,00	0,00	1,57	0,00
A4	0,00	0,00	0,16	0,10	3,69	2,92	0,49	0,00	0,00	0,00	2,44	0,00
A5	0,00	0,89	0,00	0,00	0,99	0,99	4,62	2,48	0,00	0,05	3,28	1,00
A6	0,00	0,00	0,91	0,00	1,64	1,46	1,37	3,71	0,00	0,00	2,74	0,00
A7	0,00	0,00	1,38	0,00	3,19	0,00	0,59	0,00	0,00	0,00	4,02	1,48
Spk.C	0,84	0,00	0,24	0,00	0,29	3,69	5,00	1,06	0,00	3,23	5,86	0,16
Spk.I	0,00	1,21	0,00	0,00	0,73	3,01	2,18	2,48	0,76	0,12	2,55	0,24

B

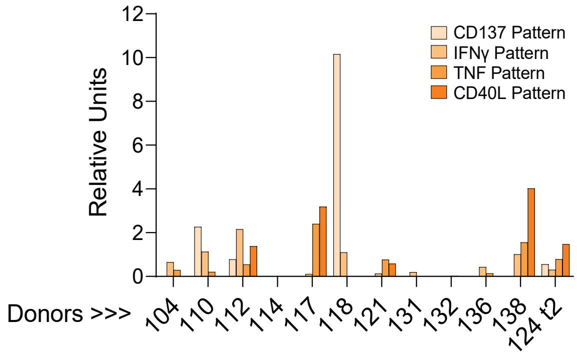

C

5 - CD137 PATTERN
CD40L+ CD137+ (All)
CD137+
CD137+ IFNγ+ (All)
CD137+ TNF+ (All)

Scale: 0–12

	104	110	112	114	117	118	121	131	132	136	138	124 t2
A1	0,00	0,37	0,00	0,47	0,13	0,48	0,28	0,43	0,44	0,24	3,19	0,10
A2	0,00	0,00	1,27	1,51	0,00	0,08	0,00	0,19	0,00	0,06	0,00	0,60
A3	0,00	0,09	4,59	2,55	0,00	0,51	0,40	0,00	0,00	0,35	0,11	0,66
A4	0,00	0,36	0,25	0,59	0,36	0,82	0,06	0,23	0,00	0,39	0,00	0,09
A5	0,00	0,05	1,38	1,01	0,00	0,44	0,31	0,22	0,00	0,16	0,00	0,00
A6	0,00	0,00	1,68	0,09	0,14	0,70	0,00	1,19	0,67	0,00	0,01	0,20
A7	0,13	1,42	1,89	0,19	0,00	0,49	0,00	0,09	0,00	0,18	0,16	0,17
Spk.C	1,56	11,95	0,78	1,41	2,75	2,38	10,58	7,20	0,00	1,70	1,91	2,95
Spk.I	0,97	4,70	0,00	0,34	2,92	0,53	8,65	3,12	0,20	2,16	2,17	1,81

6 - CD40L PATTERN
CD40L+ TNF+ (All)
CD40L+
CD40L+ IFNγ+ (All)
CD40L+ CD137+ (All)

Scale: 0–12

	104	110	112	114	117	118	121	131	132	136	138	124 t2
A1	0,00	0,17	0,00	0,00	1,08	0,94	1,72	0,24	0,31	0,56	2,57	0,00
A2	0,00	0,14	1,99	0,10	0,02	0,40	0,00	0,00	0,12	0,00	0,18	1,17
A3	0,00	0,00	1,07	0,04	0,62	0,58	0,00	0,32	0,15	0,08	0,99	0,36
A4	0,00	0,37	0,07	0,00	2,23	2,41	0,08	0,36	0,00	0,00	0,20	0,29
A5	0,00	0,00	1,30	0,00	0,00	0,24	0,00	0,41	0,00	0,55	0,00	0,09
A6	0,00	0,48	0,03	0,00	0,28	1,85	0,00	2,12	0,07	0,00	0,06	0,29
A7	0,00	3,83	1,31	0,00	0,52	0,29	0,00	0,09	0,00	0,06	0,72	1,17
Spk.C	1,66	9,45	3,61	0,46	3,43	2,62	6,69	5,10	0,00	2,75	3,04	2,56
Spk.I	0,63	6,25	0,42	0,47	3,09	0,00	7,50	1,82	1,65	2,30	2,96	1,89

7 - TNF PATTERN
TNF+
INFγ+ TNF+ (All)

Scale: 0–12

	104	110	112	114	117	118	121	131	132	136	138	124 t2
A1	0,00	3,26	0,00	0,45	1,05	0,00	0,00	1,54	8,00	0,00	2,40	0,00
A2	0,00	0,79	0,00	2,27	0,39	0,00	0,34	0,22	0,00	0,18	0,00	0,23
A3	0,00	2,33	0,00	2,64	1,72	0,00	0,00	0,02	0,19	0,36	0,57	0,30
A4	0,00	4,23	0,00	1,30	1,33	1,07	0,02	0,57	0,00	0,33	0,00	0,00
A5	0,00	1,26	0,34	1,85	0,00	0,00	1,25	0,22	0,00	0,00	0,00	0,00
A6	0,00	1,21	0,00	1,26	0,53	0,00	0,89	1,12	0,46	0,00	0,50	0,00
A7	1,37	5,13	0,00	1,26	0,60	0,00	0,00	0,01	1,35	0,00	0,82	0,53
Spk.C	2,35	5,41	0,00	2,60	2,04	0,31	8,24	6,30	0,34	0,78	0,13	0,75
Spk.I	1,90	5,99	0,00	0,78	4,41	0,39	7,42	2,42	0,15	0,90	1,29	0,84

Supplementary Figure S4. Hit identification by polyfunctionality analysis. (A) Pools identified by Reactivity Score (RS, upper section) and polyfunctionality (Polyf., lower section) analyses. Positivity thresholds for the Reactivity Score and the polyfunctionality analysis were set at 0.3 RS units and 0.01% (total double positive events above background $\geq$ 10 gated events), respectively. The pool uniquely identified by the polyfunctionality analysis is marked in white (A4, donor HD 104). (B) Distribution of triple (+++), double (++), and single (+) positive events for IFNγ, TNF, and CD137. The degree of polyfunctionality for the IFNγ gate and the TNF gate is shown for each pool. Donor's ID is shown in yellow.

A

	HD 104	HD 110	HD 112	HD 114	HD 117	HD 118	HD 121	HD 124	HD 131	HD 132	HD 136	HD 138
RS analysis	A1		A1		A1	A1	A1	A1	A1			A1
		A2	A2	A2	A2	A2	A2	A2	A2			A2
		A3	A3	A3	A3	A3	A3	A3	A3			A3
		A4	A4	A4	A4	A4	A4	A4	A4	A4		A4
		A5	A5	A5	A5	A5	A5	A5	A5		A5	A5
			A6	A6	A6	A6	A6		A6	A6		A6
	A7	A7	A7		A7	A7	A7	A7			A7	A7
Polyf. analysis												A1
				A2	A2	A2						
			A3	A3	A3							
	A4			A4		A4						
			A5	A5		A5	A5		A5			A5
				A6	A6	A6			A6	A6		
	A7		A7			A7					A7	

B

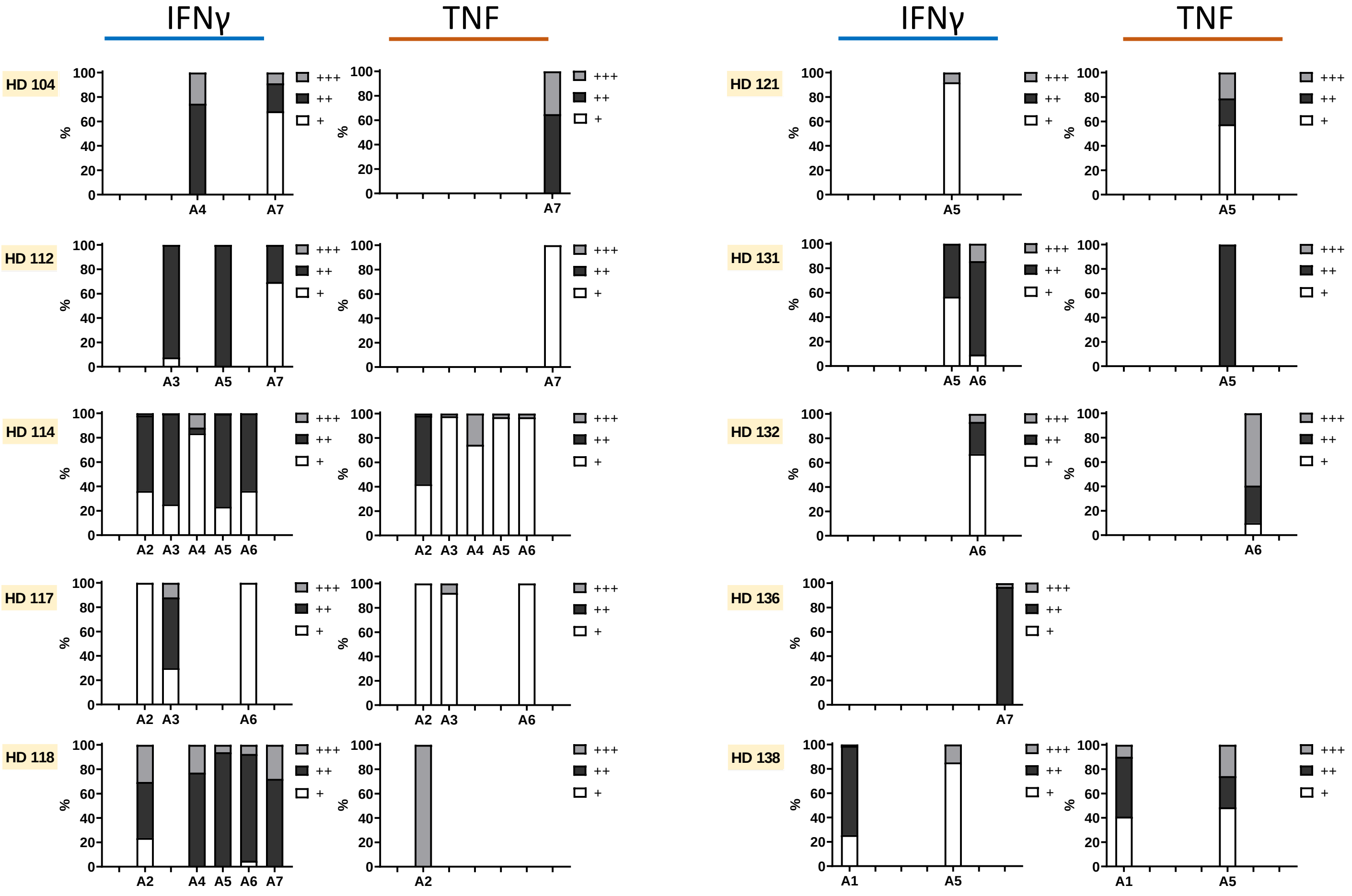

Supplementary Table S3. AIMs-defined T cell subpopulations analyzed in the study. The sixteen partially overlapping and independently quantified T cell populations used in the study are shown. These populations were defined for both the $CD4^+$ and $CD8^+$ T cell subsets.

Marker(s)	T Cell Population Designation	Counted Events
IFNγ	$IFN\gamma^+$	Single positive
TNF	TNF^+	Single positive
CD137	$CD137^+$	Single positive
CD40L	$CD40L^+$	Single positive
IFNγ and TNF	$IFN\gamma^+$ TNF^+	Double positive
IFNγ and CD137	$IFN\gamma^+$ $CD137^+$	Double positive
IFNγ and CD40L	$IFN\gamma^+$ $CD40L^+$	Double positive
TNF and CD137	TNF^+ $CD137^+$	Double positive
TNF and CD40L	TNF^+ $CD40L^+$	Double positive
CD137 and CD40L	$CD137^+$ $CD40L^+$	Double positive
IFNγ and TNF	$IFN\gamma^+$ TNF^+ All	Double positive + Single positive
IFNγ and CD137	$IFN\gamma^+$ $CD137^+$ All	Double positive + Single positive
IFNγ and CD40L	$IFN\gamma^+$ $CD40L^+$ All	Double positive + Single positive
TNF and CD137	TNF^+ $CD137^+$ All	Double positive + Single positive
TNF and CD40L	TNF^+ $CD40L^+$ All	Double positive + Single positive
CD137 and CD40L	$CD137^+$ $CD40L^+$ All	Double positive + Single positive
